# Mannose 6‐phosphonate labelling: A key for processing the therapeutic enzyme in Pompe disease

**DOI:** 10.1111/jcmm.14516

**Published:** 2019-07-10

**Authors:** Anastasia Godefroy, Morgane Daurat, Afitz Da Silva, Ilaria Basile, Khaled El Cheikh, Catherine Caillaud, Sabrina Sacconi, Benedikt Schoser, Henry‐Vincent Charbonné, Magali Gary‐Bobo, Alain Morère, Marcel Garcia, Marie Maynadier

**Affiliations:** ^1^ IBMM, CNRS, ENSCM, University of Montpellier Montpellier France; ^2^ NanoMedSyn Montpellier France; ^3^ Biochimie Métabolique et Protéique, AH‐HP, Hôpital Necker Enfants‐Malades and Inserm U1151, Institut Necker Enfants Malades Université Paris‐Descartes Paris France; ^4^ Service Système Nerveux Périphérique, Muscle et SLA, Centre Hospitalier Universitaire de Nice, Centre National de la Recherche Scientifique, Institut National de la Santé et de la Recherche Médicale, Institute for Research on Cancer and Aging of Nice Université Côte d'Azur Nice France; ^5^ Department of Neurology Friedrich‐Baur‐Institute, Ludwig‐Maximilians University Munich Munich Germany

**Keywords:** acid phosphatases, acid α‐glucosidase, enzyme replacement therapy, intracellular processing, lysosomal storage disease, mannose 6‐phosphate receptor

## Abstract

In the search of a better enzyme therapy in Pompe disease, the conjugation of mannose 6‐phosphonates to the recombinant enzyme appeared as an enhancer of its efficacy. Here, we demonstrated that the increased efficacy of the conjugated enzyme is partly due to a higher intracellular maturation because of its insensitiveness to acid phosphatases during the routing to lysosomes.

## 1 INTRODUCTION

In Pompe disease, the current enzyme replacement therapy based on a human recombinant acid α‐glucosidase (rhGAA) naturally bearing mannose 6‐phosphate (M6P) has a limited efficacy.^1^, ^2^, ^3^, ^4^ We recently proposed a conjugation of rhGAA with a phosphonate analogue of mannose 6‐phosphate (called AMFA) which enables never‐before seen improvements on walking ability and on musculoskeletal health in the aged Pompe mouse model.^5^, ^6^ Here, we hypothesized that this new therapeutic efficacy is not solely due to a better internalization via M6P receptor pathway and we investigated the enzyme processing. The intracellular maturation of rhGAA (110 kD) is complex and consists in successive proteolyses up to an endosomal 95 kD intermediate form, a 76 kD active form and a 60‐70 kD lysosomal mature form (Scheme S1).^7^, ^8^ The 76 and 60‐70 kD GAA species show a 7‐10‐fold increased activity.^7^ Bali et al^8^ clearly established on patient biopsies that the overall activity was correlated with the presence of the 60‐70 kD form.

Secondly, since an overexpression of acid phosphatases has been observed in several lysosomal storage disorders (LSD)^9^, ^10^ we also analysed the involvement of lysosomal acid phosphatases ACP2 and ACP5 in rhGAA maturation.

## 2 MATERIALS AND METHODS

See Supporting Information for detailed description.

## 3 RESULTS

### 3.1 AMFA grafting on rhGAA enables in vitro and in vivo complete enzyme processing

**Figure 1:**
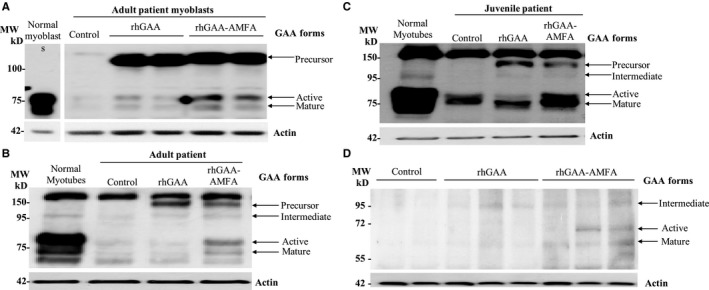
Uptake and maturation of rhGAA and rhGAA‐AMFA in myoblasts and myotubes of Pompe patients (P12, P14) and in quadriceps of treated Pompe mice. A, P12 myoblasts. B, C, myotubes differentiated from myoblasts of adult (P12) and juvenile (P14) patients were incubated with 50 nmol/L rhGAA, rhGAA‐AMFA or with vehicle (Control) for 8 h (myoblasts) or 48 h (myotubes) in culture medium. The cell extracts (5 µg) were analysed by Western blot using human GAA or actin antibodies. D, Maturation of rhGAA and rhGAA‐AMFA in quadriceps of aged Pompe mice. The tests were performed on 10‐month‐old mice treated with 5 mg/kg/week of rhGAA or rhGAA‐AMFA or by vehicle (Control), during 13 weeks. The maturation of the enzymes in quadriceps on aged Pompe mice is studied by Western blot on 20 µg tissue extract using human GAA or actin antibodies. Black arrows indicate respectively 110 kD (inactive precursor), 95 kD (inactive intermediary), 76 kD (active intermediary) and 60‐70 kD GAA (mature active) forms and actin is a control for total protein loading

A high uptake of 110 kD GAA precursor was observed in cultured myoblasts from adult Pompe patients for both rhGAA and rhGAA‐AMFA treatments (Figure 1A); however, 76 kD form (Figure S1A) and 60‐70 kD form (Figure S1B) are 3‐ and 5‐fold more important for rhGAA‐AMFA treated cells. As expected intracellular GAA activity was also significantly increased for rhGAA‐AMFA as compared to rhGAA (Figure S1C). Similar results were found on different myoblasts (Figure S1D‐G). GAA genetic mutations of the corresponding primary cultured cells are listed in Table S1.

We then differentiated myoblasts into myotubes according to the technique described by Nascimbeni et al 2012.^11^ In myotubes, the 76 kD and 60‐70 kD forms were significantly increased by rhGAA‐AMFA treatment (4.1‐ and 2.2‐fold) as compared to rhGAA (Figure 1B‐C).

We also analysed the enzyme maturation in 10‐month‐old Pompe mice treated weekly with 5 mg/kg rhGAA, rhGAA‐AMFA or vehicle during 3 months. While no specific band was detected in the control Pompe tissue, the 95 kD intermediary form was detected after rhGAA treatment (Figure 1D). In mice injected with rhGAA‐AMFA, the 95 kD form, the 76 kD active form and/or the 60‐70 kD mature form were observed. This higher maturation was also associated with a gain in GAA activity in muscle biopsies (data not shown). Together, these results indicate that AMFA conjugation increases rhGAA maturation both in patient cultured cells and in aged Pompe mice.

### 3.2 Impact of acid phosphatases in GAA processing

**Figure 2:**
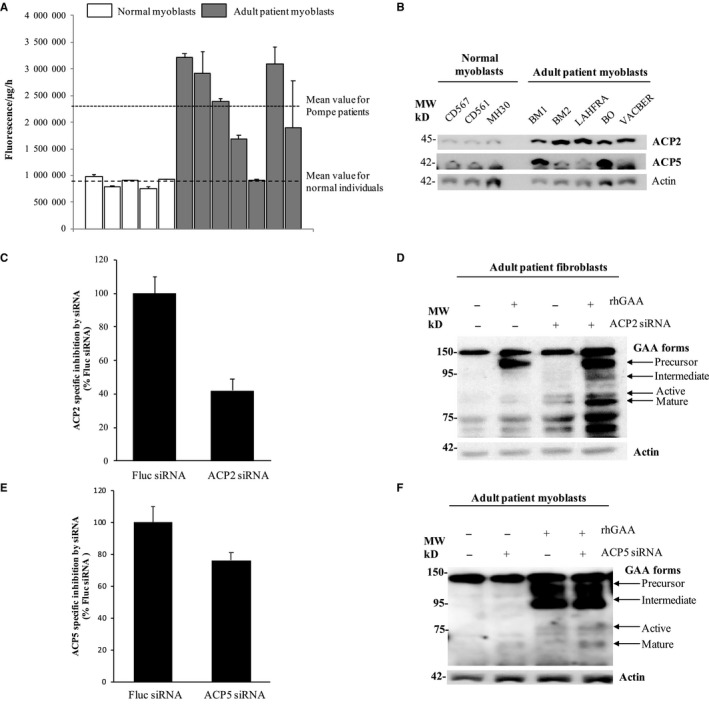
Overexpression of acid phosphatases and activation of rhGAA maturation by silencing phosphatases in cells from Pompe patients. A, The phosphatase activity was respectively measured in P1, P2, P4, P5, P6 myoblasts from healthy individuals and P7, P8, P9, P10, P11, P12, P13 myoblasts from adult Pompe patients. The quantification was assayed on cell extracts (10 µL) using a fluorescent substrate 6,8‐difluoro‐4‐methylumbelliferyl phosphate (DiFMUP) (n = 2). B, Cell extracts (5 µg) of myoblasts from different origins were analysed by Western blots using anti‐human ACP2, ACP5 or actin antibodies (used as loading control). C, D, With specific siRNA, ACP2 expression was inhibited in P15 fibroblasts. E, F, In P12 myoblasts, ACP5 expression was modulated through siRNA treatment . Western blot analysis of lysates from cells incubated with 50 nmol/L rhGAA for 8 h preceded or not by 48 h siRNA treatment. Lysates were immunoblotted with antibody against human GAA and actin as loading control (n = 2)

The main difference between AMFA and Mannose 6‐Phosphate is the replacement of the phosphate moiety by a phosphonate group insensitive to phosphatase in contrast to M6P. In agreement with a previous study,^9^ we found that acid phosphatases activity is significantly increased in Pompe patient myoblasts (264 ± 96%) as compared to normal myoblasts (value set as 100 ± 11%, *P* < 0.005 Student's *t* test) (Figure 2A). As such phosphatases could be involved in M6P signal deterioration on rhGAA, we demonstrated that the inhibition of overexpressed phosphatases with sodium fluoride or β—glycerophosphate allowed the processing of rhGAA into 76 kD and 60‐70 kD active forms (Figure [Link]).

Expressions of ACP2 and ACP5, two major acid phosphatases involved in M6P dephosphorylation^9^ were assessed (Figure 2B). ACP2 is overexpressed in almost all Pompe disease samples, whereas few ones overexpressed ACP5.

The inhibition with specific siRNAs of 58% ACP2 and 24% ACP5 expression (Figure 2C‐E) was sufficient to enhance rhGAA maturation (Figure 2D‐F) and partially that of the endogenous deficient enzyme.

## 4 DISCUSSION

In this report, significant differences in the processing of rhGAA‐AMFA and rhGAA are presented. Although both enzymes are well internalized, only rhGAA‐AMFA undergoes cleavage to 76 kD active and 60‐70 kD mature forms in primary cultures of fibroblasts, myoblasts and myotubes from Pompe patients and in aged Pompe mice after 3‐month treatment. These data establish that the protease machinery necessary for enzymatic maturation is still functional in both adult patients and aged Pompe mice which were previously considered as ERT refractory.^4^

Then, we considered the role of phosphatases in GAA maturation. In LSD, several reports evidence an overexpression of lysosomal acid phosphatases. As already demonstrated for lysosomal enzymes in cancers,^12^, ^13^ we supposed that overexpressed phosphatases can overflow from lysosomes to nearby endo‐lysosomal vesicles. Such abnormally localized phosphatases could prevent GAA‐M6PR complexing before the enzyme reaches the lysosomes and thus impair enzyme endo‐lysosomal maturation. Our present analysis indicates an increase of the acid phosphatases activity and more specifically, an elevation of ACP2 and ACP5. Using general inhibitors and specific siRNAs, we obtained a partial phosphatases inhibition sufficient to allow the formation of rhGAA active and mature forms. Although different cell types remain to be evaluated, these data already evidence that acid phosphatases overexpression prevents rhGAA processing.

The outcome of the unmatured rhGAA was not investigated here. However, aberrant localization of rhGAA into autophagosomes could be hypothesized.^14^

In conclusion, the higher therapeutic efficacy of rhGAA‐AMFA observed in vitro and in vivo 5, 6 is associated with an increase of enzyme maturation. Altogether, these data suggest that AMFA targeting may represent a potential therapeutic advantage for Pompe disease and also for other LSD which overexpress acid phosphatases.

## CONFLICT OF INTEREST

The authors declare that they have no conflicts of interest with the contents of this article.

## Data Availability

The data that support the findings of this study are available from the corresponding author upon reasonable request.
