# Supplementary material for: Mannose 6‐phosphonate labelling: A key for processing the therapeutic enzyme in Pompe disease
Source: J Cell Mol Med. 2019 Jul 10;23(9):6499–503. doi: 10.1111/jcmm.14516 (PMC6714136; doi:10.1111/jcmm.14516)
Supplement: Supplementary file 1 [file JCMM-23-6499-s001.docx]

# SUPPORTING INFORMATIONS

**
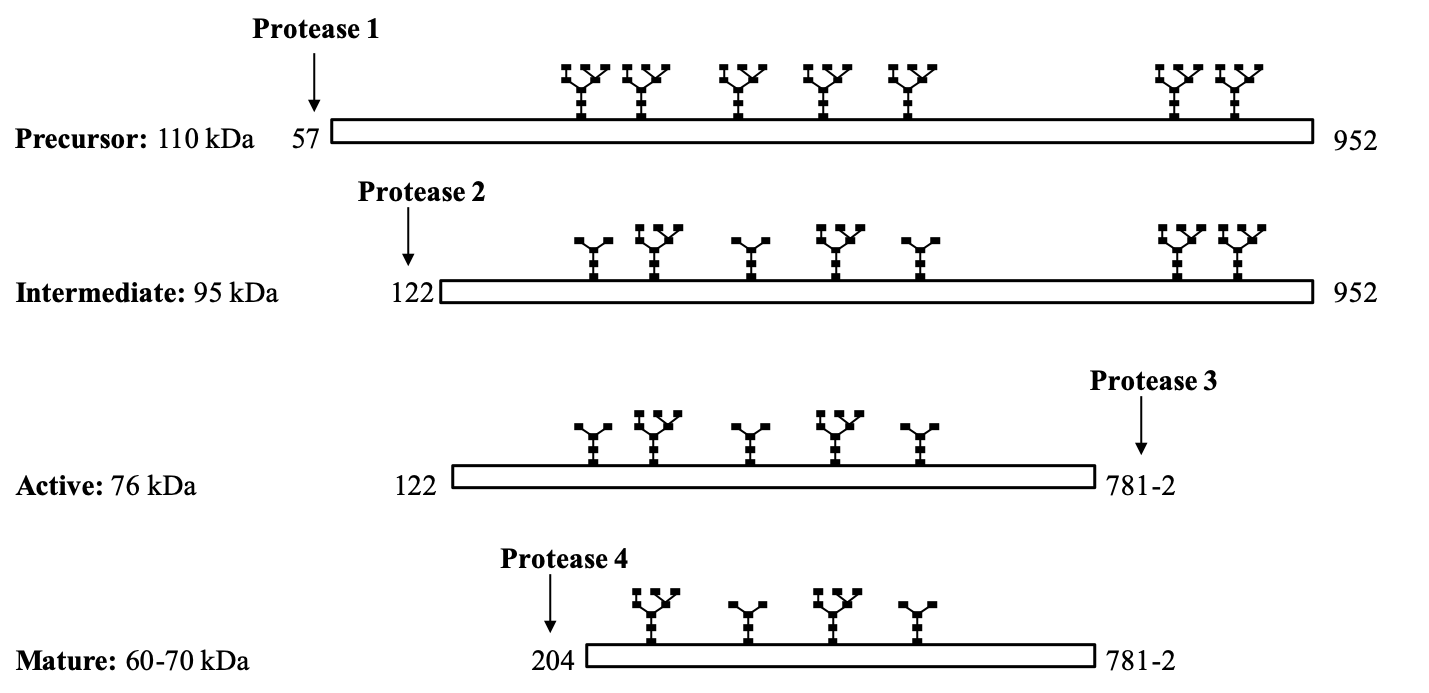
**

**Scheme S1. Intracellular maturation of GAA under physiological conditions.** Several successive cleavages of the inactive 110 kDa precursor GAA occur in the endolysosomal routing leading to the formation of an intermediate 95 kDa form, then an active 76 kDa form and finally the mature 60-70 kDa form in lysosomes (modified from [12] ).

## *MATERIALS & METHODS*

## *Materials*

Primary cultures of myoblasts from 15 individuals (referred as P1 to 15) were provided by different biobanks. Normal myoblasts (referred as P1 to P6) were supplied by Dr. G. Carnac (Inserm U1046-UMR CNRS 9214, Montpellier, France) and the Reference Center for Neuromuscular Diseases and ALS of Nice University Hospital (Nice, France).

## Pompe adult myoblasts (referred as P7 to P13) were provided by the Muscle Tissue Culture Collection (Munich, Germany), the Reference Center for Neuromuscular Diseases and ALS of Nice University Hospital (Nice, France) and the CBC Biotec Biobank BB-0033-00046 (Lyon, France). P14, a primary culture of myoblasts from a Pompe juvenile patient, was obtained from the Telethon Network of Genetic Biobanks [1] (Milan, Italy). P15 corresponds to fibroblasts from Pompe adult patient which were kindly provided by Dr. C. Caillaud (Necker Hospital Paris, France). The GAA gene alterations identified in Pompe patients from which the primary cultures were established are summarized in Table S1.

| **Table S1.** *GAA* gene mutations in the Pompe patients used in this study | | |
| --- | --- | --- |
| **Patient** | **Allele 1** | **Allele 2** |
| P7 | c-32-13T>G | c.925G>A (p.Gly309Arg) |
| P8 | c-32-13T>G | c.1075G>A (p.Gly359Arg) |
| P9 | c-32-13T>G | c.1438-1G>C |
| P10 | c.-32-13T>G | c.1396del (p.Val466Phefs*11) |
| P11 | c.-32-13T>G | c.2738C>G (p.Pro913Arg) |
| P12 | c-32-13T>G | c.1927G>A (p.Gly643Arg) |
| P13 | c.119G>A (p.Arg40Gln) | c.1497G>A (p.Trp499*) |
| P14 | c.-32-13T>G | c.2237G>A (p.Trp746*) |
| P15 | c.-32-13T>G | c.2104C>T (p.Arg702Cys) |

## The rhGAA used was alglucosidase-alfa (Myozyme®) manufactured by Sanofi-Genzyme and recovered from excess material of the reconstituted commercial product used in patients. Pompe mice were a kind gift from Pr. Nina Raben [2] and were housed and cared according to protocols approved by the Languedoc-Roussillon ethical committee CEEA-LR-36 with agreement N4987-2016101116132551.

## *Cell culture*

Cells were cultured in a humidified atmosphere containing 5% CO_2_ at 37°C. The myoblasts of Pompe disease patients and healthy persons were cultured in Ham F10 medium plus 20% fetal bovine serum (FBS), 1% insulin, 25 ng/mL FGF, 10 ng/mL EGF and 1% penicillin/streptomycin on collagen coated surface. In myotube experiments, successive steps of the differentiation of adult patient myoblasts are required. Myoblasts at confluence were induced to differentiate by lowering the FBS concentration to 2%. Cells were maintained in differentiation medium for 5 days. The myotubes obtained after 7 days of differentiation acquired contractile capacities (data not shown) and are therefore representative of the most abundant cells of skeletal muscles. For fibroblasts, they were cultured in DMEM supplemented with 10% FBS and 1% penicillin/streptomycin.

## *Chemical conjugation of AMFA onto rhGAA*

## The rhGAA was coupled with AMFA according to the method previously described [10] . Briefly, rhGAA was oxidized with 5 mM NaIO_4_ for 30 min at 4°C in the dark. Oxidation reaction was stopped by addition of 2% glycerol. Oxidized enzyme was purified on a G-10 Sepharose column (GE Healthcare) according to manufacturer’s instructions. AMFA was then grafted on rhGAA at a ratio of 300 equivalents per mole of enzyme during 2 h at 37°C in the dark under agitation. After conjugation reaction, rhGAA-AMFA was dialyzed against 25 mM sodium phosphate buffer (pH 6.2) containing 1% mannitol and 0.005% Tween 80 for 18 h at 4°C. The samples were aliquoted and stored at -20°C until used. The number of AMFA, determined using MALDI-TOF mass spectrometry from 5 different coupling reactions, is approximately of 5.3 ± 1.8 mol AMFA per mol enzyme.

## *Western blot analysis*

To study rhGAA-AMFA maturation, extracts from Pompe adult myoblasts were subjected to Western blot analysis. The cells were washed 3 times, harvested in PBS and lysed by 3 freeze-thaw cycles in buffer containing 50 mM HEPES, 150 mM NaCl, 1 mM EDTA, 2.5 mM EGTA, 0.1% Tween 20, 10% glycerol, 1 mM NaF, 1 mM NaNO_3_, 10 mM glycerophosphate and protease inhibitors (dilution 1:25 Complete, Roche Diagnostics). The lysates were precleared by centrifugation at 10,000 g for 15 min at 4°C. Samples were tested for protein concentration by the Bradford method and equal amounts (5 or 20 µg) of cell extract were resolved by 12% SDS-PAGE. After blotting the gel into PVDF membrane, proteins were detected by probing with anti-human GAA (GeneTex), ACP2 (Santa Cruz Biotechnology) or ACP5 (Abcam) incubated with peroxidase-conjugated secondary antibodies (Amersham). Immunoreactive proteins were then analyzed by ECL detection system (Amersham). Actin detected by a monoclonal mouse antibody provided by Dr N. Bettache (CNRS UMR5247, Montpellier, France) was used as the loading control. Quantifications of the immunoreactive GAA forms were performed on unsaturated films with low exposures using Image J software.

## *Uptake assays*

After enzyme treatment in triplicate for the indicated times at 37°C, cells were washed 3 times with PBS and lysed by sonication in GAA assay buffer (0.2 M C_2_H_3_NaO_2_, 0.4 M KCl, pH 4.3) containing 0.1% Triton X-100. The lysates were centrifuged at 14,000 g for 10 min at 4°C. Samples were tested for protein concentration and enzymatic activity was measured by using the fluorescent substrate 4-methylumbelliferyl-α-D-glucopyranoside (4-MUG, Sigma Aldrich). Cell lysates were incubated with 160 µM 4-MUG diluted in GAA assay buffer for 3 h at 37°C. The reaction was stopped by addition of 0.4 M glycine buffer pH 10.4 and the fluorescence was read with 355 nm excitation and 460 nm emission filters with a PerkinElmer 1420 VICTOR 2 microplate reader.

## *Total acid phosphatases activity quantification*

Cells were cultured in 6-well plates and lysed in 0.2 M C_2_H_3_NaO_2_, 0.4 M KCl, 0.1% Triton pH 4.3 and sonicated 3×6 s. The lysates were centrifuged at 14,000 g for 10 min at 4°C. Samples were assayed for protein concentration and the activity of phosphatases was determined using 6,8-difluoro-4-methylumbelliferyl phosphate (DiFMUP) (Molecular Probes) as a substrate. Extracts (5 μL) were incubated in 100 mM sodium acetate buffer pH 5.5 and 0.1 mM DiFMUP for 30 min at room temperature. Fluorescence intensity was monitored with a 355 nm excitation and a 460 nm emission filters with a PerkinElmer 1420 VICTOR 2 microplate reader.

***Acid phosphatases inhibition***

Cells were incubated with 50 nM enzymes and phosphatase inhibitors 2 mM NaF or 2.5 mM beta-glycerophosphate for 48 h at 37°C in RPMI medium enriched with 10% heat-inactivated calf serum. After incubation, cells were washed three times with PBS and stocked at -20°C until Western blot analysis.

***siRNA silencing***

Small interfering RNA (siRNA) duplex oligoribonucleotides for human acid phosphatases 2 (ACP2) and tartrate-resistant acid phosphatase (TRAP, ACP5) were synthesized by Santa Cruz Biotechnology, INC (accession number: sc-96327 and sc-44164). ACP2 siRNA and TRAP siRNA are a pool of 3 target-specific 19-25 nucleotides designed to knock down gene expression. Firefly luciferase-specific siRNA (siRNA Fluc) was used as controlled and purchase by Eurogentec. siRNA Fluc sequences were as followed: sense 5’-CUUACGCUGAGUACUUCGA55-3’ and anti-sense 5’UCGAAGUACUCAGCGUAAG-3’. Cells were allowed to grow on 6-well plates until reaching 50-70% confluency in appropriate culture medium. Cells were then transfected using, for one well, 50 pmol of siRNA and 8 µL of INTERFERin® (PolyplusTransfection) reagent in Opti-MEM^TM^ medium and incubated 4 h. Following incubation, standard growth medium was added and FCS equilibrated at 10% in each well. Forty-eight h after transfection, wells were washed 2 times with PBS and treated with 50 nM enzymes or vehicle for 8 h in DMEM supplemented with 10% FCS. After incubation, cells were washed 3 times with PBS and plates were stored at -20°C until used.

***Statistical analysis***

The statistical analysis was performed using either the Student's test or the Newman-Keuls test for repeated measures. A probability value of p< 0.05 was considered statistically significant.

REFERENCES

[1] **Filocamo M, Mazzotti R, Corsolini F, et al.** Cell Line and DNA Biobank From Patients Affected by Genetic Diseases. *Open Journal of Bioresources* 2014; 1; e2. (methods)

[2] **Raben N, Nagaraju K, Lee E, et al.** Targeted disruption of the acid alpha-glucosidase gene in mice causes an illness with critical features of both infantile and adult human glycogen storage disease type II. *J. Biol. Chem.* 1998; 273; 19086–92(.Mettre in methods)

## *ADDITIONAL RESULTS*

***Maturation of rhGAA and rhGAA-AMFA in myoblasts of adult Pompe patien***t.

**
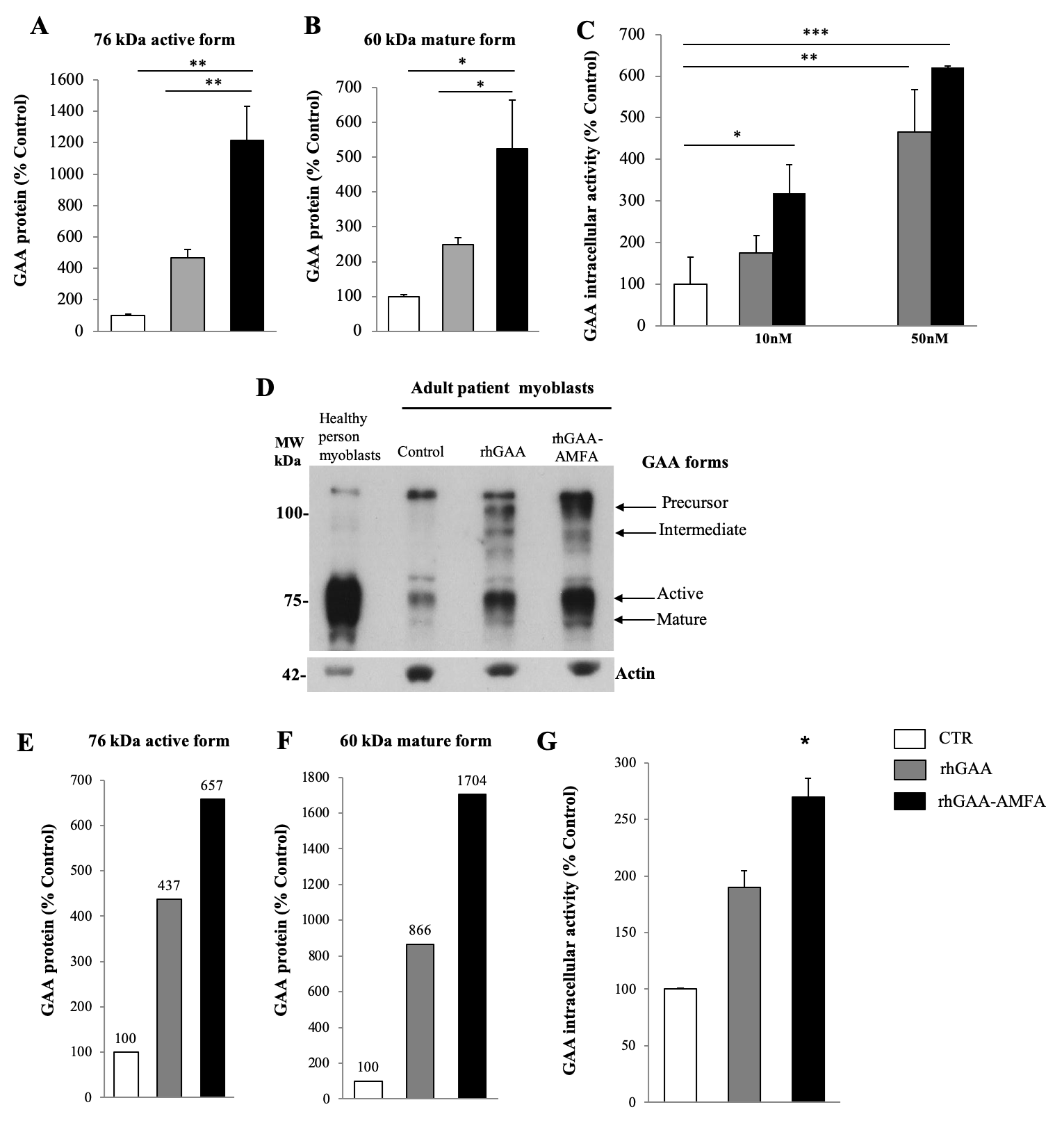
**

**Figure S1:** **Maturation of rhGAA and rhGAA-AMFA in myoblasts of Pompe patient P12 (from A to C) and P7 (from D to G).** Myoblasts were incubated with 50 nM rhGAA, rhGAA-AMFA or with vehicle (Control) for 8 h (P12) or 4 h (P7) in medium enriched with 10% human serum. (A, B) Quantification with Image J software of 76 kDa and 60-70 kDa GAA protein forms corrected by actin expression. Control cells are considered as 100%. (n=2) (C) GAA catalytic activities of myoblasts treated 3 h with vehicle or 10 nM or 50 nM rhGAA or rhGAA-AMFA. The tests were performed in cell lysates using synthetic substrate 4-MUG. Data are expressed as a percentage of vehicle treated cell activity (n=2), *p<0.05, **p<0.01 and ***p<0.001 (Newmann-Keuls multiple comparison test). **(**D) The cell extracts (5 µg) were analyzed by Western blots using human GAA or actin antibodies. Black arrows indicate respectively 110 kDa (inactive precursor), 95 kDa (inactive intermediate), 76 kDa (active intermediary) and 60-70 kDa GAA (mature active) forms. Actin is a control for total protein loading. (E, F) Quantification of GAA protein forms performed as in (A,B). (G) GAA activities in P7 myoblasts incubated for 3 h with 20 nM enzyme treatment (n=2), *p<0.05 vs control (Newmann-Keuls multiple comparison test).


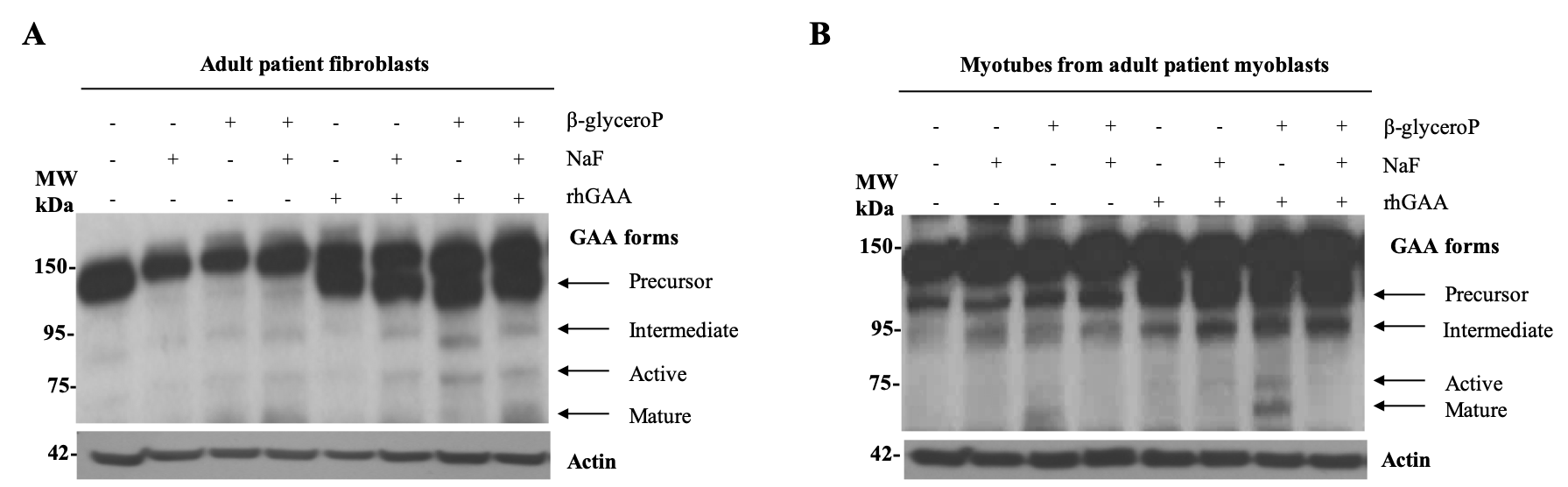


**Figure S2: Activation of rhGAA maturation by phosphatase inhibitors in adult Pompe fibroblasts and myotubes.** The internalization and processing of rhGAA in P15 adult fibroblasts (A), myotubes from P9 (B) was measured in the presence or absence of phosphatase inhibitors NaF or beta-glycerophosphate. The enzymes (50 nM) were incubated for 48 h and the intracellular maturation was detected by Western blots using an anti-human GAA antibody. Actin was used as a control for total protein loading.

The combination of two inhibitors did not show additive effect on GAA maturation in fibroblasts and decreased the effect of beta-glycerophosphate in myotubes. These results indicate that the cellular acid phosphatases could play a repressive role in the maturation of rhGAA. In fibroblasts, the loss of the M6P signal on rhGAA would probably happen before the first proteolytic cleavage of the 110 kDa precursor since the 95 kDa is not detected. In myotubes and myoblasts the 95 kDa form is detected suggesting that phosphate group of M6P was removed later.
